# Editorial: Advances in chronic ischemic cerebrovascular disease: diagnosis and management

**DOI:** 10.3389/fneur.2024.1440175

**Published:** 2024-11-29

**Authors:** Nico Sollmann, Yu Lei, Michael E. Sughrue

**Affiliations:** ^1^Department of Diagnostic and Interventional Radiology, University Hospital Ulm, Ulm, Germany; ^2^Department of Diagnostic and Interventional Neuroradiology, School of Medicine, Klinikum rechts der Isar, Technical University of Munich, Munich, Germany; ^3^TUM-Neuroimaging Center, Klinikum rechts der Isar, Technical University of Munich, Munich, Germany; ^4^Department of Neurosurgery, Huashan Hospital, Fudan University, Shanghai, China; ^5^National Center for Neurological Disorders, Shanghai, China; ^6^Department of Neurological Surgery, Columbia University, New York, NY, United States

**Keywords:** brain imaging methods, cerebrovascular disease, multi-modal imaging, magnetic resonance imaging, stroke

Ischemic cerebrovascular diseases relate to a variety of large vessel and small vessel angiopathies, which are common causes of ischemic stroke and cognitive dysfunction, especially in the elderly population (1–5). Although complex pathophysiological processes and high rates of morbidity make ischemic cerebrovascular diseases challenging, new clinical evidence continues to emerge for an improved understanding and treatment. Currently, basic and clinical research focuses on a variety of approaches to accelerate the understanding of the disease, improve patient care and monitoring, and, ultimately, reduce patient morbidity and mortality, with advanced neuroimaging techniques being used for biomarker development and testing in this regard (6–10). Furthermore, recent major research trajectories focused on aspects of advanced brain function, given that chronic ischemic conditions are also increasingly recognized as relevant risk factors for clinically silent brain infarctions and cognitive impairment (11–14).

Two studies of the Research Topic investigated patients with moyamoya disease (MMD) (Yu et al., Cao et al.). Specifically, MMD is a cerebrovascular disorder with typically progressive steno-occlusive disease, predominantly involving the bilateral intracranial internal carotid artery (ICA) and the proximal branches (15, 16). The disease is mainly observed in Japan, whereas the incidence in other parts of the world is rather low (15, 17, 18). The disease can entail symptoms related to brain ischemia (e.g., stroke) and/or related to compensatory mechanisms that can occur in response to ischemia (e.g., hemorrhage from fragile collateral vessels) (15, 16). In the study by Cao et al., hemodynamic abnormalities in 42 patients with MMD were examined before and after intervention by combined revascularization (i.e., surgery involving a direct superficial temporal artery-middle cerebral artery bypass and an indirect encephalo-arterio-synangiosis bypass) using intraoperative FLOW800, a specialized software utilizing indocyanine green video-angiography to analyze data and generate color delay mapping. The courses of parameters and associations between intraoperative FLOW800 and color Doppler ultrasonography (CDUS) measurements were evaluated, showing that both the intraoperatively measured delay time (i.e., the time interval between 0 and 50% of maximum fluorescence intensity for FLOW800 analysis) and rise time (i.e., the time interval between 10 and 90% of the maximum fluorescence intensity for FLOW800 analysis) substantially decreased in the recipient artery and vein (Cao et al.). Microvascular transit time from intraoperative assessment was significantly reduced after surgery, and all postoperatively collected parameters from CDUS measurements exhibited significant changes in comparison to the preoperative state (Cao et al.). Significant associations between microvascular transit time, resistance index, and pulsatility index were observed (Cao et al.). Based on the finding that hemodynamic outcomes of the donor and recipient arteries demonstrated significant changes following bypass surgery, the authors concluded that the parameter of time may be a more precise and sensitive measure of hemodynamics using the FLOW800 system (Cao et al.). Additionally, multiple hemodynamic evaluations could offer beneficial data for perioperative management in MMD (Cao et al.). In the study by Yu et al., metabolites associated with MMD were investigated by means of liquid chromatography coupled with mass spectrometry (LC-MS) to discern metabolic biomarkers in cerebrospinal fluid (CSF) samples. The patient group consisted of 16 patients with MMD, which were compared to a healthy control group of eight subjects (Yu et al.). The CSF samples were obtained during bypass surgery in the patient group and from lumbar puncture (during lumbar anesthesia) in the control group (Yu et al.). Overall, 129 differential metabolites in the CSF samples were identified when comparing the MMD patients to the healthy controls, which were associated with multiple pathways (e.g., purine metabolism and pyrimidine metabolism, among other pathways) (Yu et al.). Hence, using an LC-MS-based metabolomics approach may hold promise for enhancing the clinical diagnosis of MMD, and the identified parameters may lead to the development of novel diagnostic markers for MMD regarding the pathogenesis of the disease (Yu et al.).

Three articles published as part of the Research Topic focused on stroke regarding diagnostics or treatment (Yi et al.; Wu et al.; Wang et al.). Acute stroke is defined by an acute onset of focal neurological deficits, which can manifest due to the loss of blood supply related to ischemic or hemorrhagic stroke affecting brain territories or regions (19–21). In addition to neurological assessment, the diagnostic approach commonly includes imaging of the brain parenchyma, brain-feeding arteries, and oftentimes perfusion imaging using computed tomography (CT) or magnetic resonance imaging (MRI) (22, 23). In the study by Wang et al., a task-driven cerebral angiographic imaging (TDCAI) technique using CT perfusion images from patients with hemorrhagic or ischemic stroke was developed. Specifically, the TDCAI approach included registration and skull removal, vessel segmentation, artery-vein separation, centerline extraction, and vessel straightening as the major steps of processing (Wang et al.). The output was supplemented by diagnostic images obtained through the TDCAI approach, including CT angiography, CT venography, centerline images of the vessels of interest (i.e., ICA or Labbé vein), and straightened images of the vessels of interest (Wang et al.). Moreover, TDCAI-CT angiography exhibited arterial architecture with greater detail (as compared to conventional imaging in 10 patients with intracranial hemorrhagic stroke and 10 patients with acute ischemic stroke), thus also visualizing small terminal branches of arteries (Wang et al.). Similarly, TDCAI-CT venography provided enhanced visualization of venous structures, clearly depicting the Labbé vein, which can be linked to hemorrhagic stroke (Wang et al.). Hence, the TDCAI technique may enable the elimination of bone and soft tissue interference, improve the segregation of cerebral venous and arterial architecture, and enable the extraction of centerlines and straightened vessels of interest to aid in assessing the outflow profiles of vessels after stroke (Wang et al.). The study by Yi et al. analyzed predictors of futile recanalization in basilar artery occlusion following endovascular treatment, which represented a *post-hoc* analysis of The Trial of Endovascular Treatment of Acute Basilar-Artery Occlusion (ATTENTION). Specifically, ATTENTION aimed at evaluating the effects of endovascular thrombectomy in addition to best medical management compared to best medical management alone in patients with basilar artery occlusion (24). The current *post-hoc* analysis compared demographics, clinical characteristics, acute stroke workflow interval times, and imaging characteristics between futile recanalization and favorable recanalization, with favorable outcome being defined as a modified Rankin scale score of 0–3 at 90 days, successful reperfusion being defined as thrombolysis in cerebral infarction 2b and 3 on the final angiogram, and futile recanalization being defined as failure to achieve a favorable outcome despite successful reperfusion (Yi et al.). Using multivariate analyses to identify the predictors of futile recanalization, the authors showed among the 185 included patients (89 patients with futile recanalization and 96 patients with favorable recanalization) that older age and diabetes mellitus were independent predictors of futile recanalization (Yi et al.). Those two parameters may thus relate to poor outcomes in patients with acute basilar artery occlusion, but could not be directly modified in the context of emergency medical treatment (Yi et al.). In the retrospective study by Wu et al. that aimed to systematically identify risk factors for vascular complications during non-emergency endovascular treatment in patients with ICA occlusions, 92 patients were investigated using correlation analyses between intraoperative vascular complications and potential risk factors, followed by interaction analyses. The authors revealed that the use of non-neurology guide wires to open vessels and glycosylated hemoglobin (HbA1c) ≥ 6.5 mmol/L were significantly associated with vascular complications, with restricted cubic spline indicating that the higher the HbA1c level, the higher the risk of vascular complications (Wu et al.). Hence, the use of non-neurology guide wires for vessel opening may increase the risk of vascular complications, and preoperative assessment and management of HbA1c levels might help to reduce the incidence of intraoperative vascular complications (Wu et al.).

The article by Liu et al. reviewed the literature landscape regarding endovascular treatment options for intracranial ICA bifurcation region aneurysms. Yet, due to the complexity of such aneurysms and the considerable variability in contemporary endovascular therapy techniques, the approach to the treatment of ICA bifurcation aneurysms remains challenging and non-standardized (Liu et al.). The review discusses in detail the anatomy of the ICA bifurcation region, classification schemes, natural history, and treatment approaches for ICA bifurcation region aneurysms (Liu et al.). It also reviews techniques for identifying ICA bifurcation region aneurysms, as well as the prognosis and complications of endovascular therapy for such aneurysms (Liu et al.). The authors conclude that conventional coiling seems to be the currently preferred treatment option for ICA bifurcation region aneurysms, while in selected cases, new devices such as flow diverters and woven EndoBridge devices could also be used to effectively treat ICA bifurcation region aneurysms (Liu et al.).

To conclude, this Research Topic covered a large spectrum of diagnostic and treatment aspects regarding cerebrovascular disorders, with a focus on novel diagnostic approaches and the development of potential diagnostic markers. The published studies may have the potential to facilitate improved diagnostics in patients with diseases such as MMD or hemorrhagic or ischemic stroke, and could also contribute to an improved understanding of disease (patho)physiology and characteristics. Moreover, especially diagnostic techniques that are not widely available or are emerging have been covered, and those could provide unique or complementary information when brought into context with standard diagnostic approaches using CT or MRI. Finally, more research is necessary regarding tailoring the presented procedures to clinical needs or to accelerate the transition of experimental findings to broader applications in patients. In this regard, studies using large-scale data from patients with large vessel and small vessel angiopathies and ultimately ischemic cerebrovascular diseases can help to identify common patterns from quantitative imaging related to the diagnosis, and could also help in patient phenotyping or stratification. Regarding studies published in this Research Topic on treatment options for cerebrovascular disorders, new evidence is provided for potential risk factors and predictors of clinical outcome, which may support clinical decision-making and patient management in the future.

## References

[1] Petty GW Brown RD Jr Whisnant JP Sicks JD O'Fallon WM Wiebers DO. Ischemic stroke subtypes: a population-based study of functional outcome, survival, and recurrence. Stroke. (2000) 31:1062–8. 10.1161/01.STR.31.5.1062 10797166

[2] Shi Y Wardlaw JM. Update on cerebral small vessel disease: a dynamic whole-brain disease. Stroke Vasc Neurol. (2016) 1:83–92. 10.1136/svn-2016-000035 28959468 PMC5435198

[3] Fang MC Coca Perraillon M Ghosh K Cutler DM Rosen AB. Trends in stroke rates, risk, and outcomes in the United States, 1988 to 2008. Am J Med. (2014) 127:608–15. 10.1016/j.amjmed.2014.03.017 24680794 PMC4125206

[4] Herrington W Lacey B Sherliker P Armitage J Lewington S. Epidemiology of atherosclerosis and the potential to reduce the global burden of atherothrombotic disease. Circ Res. (2016) 118:535–46. 10.1161/CIRCRESAHA.115.307611 26892956

[5] Wardlaw JM Smith C Dichgans M. Small vessel disease: mechanisms and clinical implications. Lancet Neurol. (2019) 18:684–96. 10.1016/S1474-4422(19)30079-1 31097385

[6] Schmitzer L Kaczmarz S Gottler J Hoffmann G Kallmayer M Eckstein HH. Macro- and microvascular contributions to cerebral structural alterations in patients with asymptomatic carotid artery stenosis. J Cereb Blood Flow Metab. (2024) 2024:271678X241238935. 10.1177/0271678X241238935 38506325 PMC11418673

[7] Schmitzer L Sollmann N Kufer J Kallmayer M Eckstein HH Zimmer C. Decreasing spatial variability of individual watershed areas by revascularization therapy in patients with high-grade carotid artery stenosis. J Magn Reson Imaging. (2021) 54:1878–89. 10.1002/jmri.27788 34145686

[8] Wardlaw JM Smith C Dichgans M. Mechanisms of sporadic cerebral small vessel disease: insights from neuroimaging. Lancet Neurol. (2013) 12:483–97. 10.1016/S1474-4422(13)70060-7 23602162 PMC3836247

[9] Thomas DL Lythgoe MF Pell GS Calamante F Ordidge RJ. The measurement of diffusion and perfusion in biological systems using magnetic resonance imaging. Phys Med Biol. (2000) 45:R97–138. 10.1088/0031-9155/45/8/201 10958179

[10] Alves GS Sudo FK Alves CEO Ericeira-Valente L Moreira DM Engelhardt E. Diffusion tensor imaging studies in vascular disease: a review of the literature. Dement Neuropsychol. (2012) 6:158–63. 10.1590/S1980-57642012DN06030008 29213790 PMC5618963

[11] Lal BK Dux MC Sikdar S Goldstein C Khan AA Yokemick J. Asymptomatic carotid stenosis is associated with cognitive impairment. J Vasc Surg. (2017) 66:1083–92. 10.1016/j.jvs.2017.04.038 28712815

[12] Muller M van der Graaf Y Algra A Hendrikse J Mali WP Geerlings MI. Carotid atherosclerosis and progression of brain atrophy: the SMART-MR study. Ann Neurol. (2011) 70:237–44. 10.1002/ana.22392 21674583

[13] Wardlaw JM Sandercock PA Dennis MS Starr J. Is breakdown of the blood-brain barrier responsible for lacunar stroke, leukoaraiosis, and dementia? Stroke. (2003) 34:806–12. 10.1161/01.STR.0000058480.77236.B3 12624314

[14] Ostergaard L Engedal TS Moreton F Hansen MB Wardlaw JM Dalkara T. Cerebral small vessel disease: capillary pathways to stroke and cognitive decline. J Cereb Blood Flow Metab. (2016) 36:302–25. 10.1177/0271678X15606723 26661176 PMC4759673

[15] Scott RM Smith ER. Moyamoya disease and moyamoya syndrome. N Engl J Med. (2009) 360:1226–37. 10.1056/NEJMra0804622 19297575

[16] Smith ER Scott RM. Moyamoya: epidemiology, presentation, and diagnosis. Neurosurg Clin N Am. (2010) 21:543–51. 10.1016/j.nec.2010.03.007 20561502

[17] Yonekawa Y Ogata N Kaku Y Taub E Imhof HG. Moyamoya disease in Europe, past and present status. Clin Neurol Neurosurg. (1997) 99(Suppl.2):S58–60. 10.1016/S0303-8467(97)00042-5 9409407

[18] Liu H Fukasawa T Anno T Takeuchi M Shimazaki S Yang T. Incidence, prevalence, and treatment of Moyamoya disease in Japan: a population-based descriptive study. J Stroke Cerebrovasc Dis. (2024) 2024:107770. 10.1016/j.jstrokecerebrovasdis.2024.107770 38768667

[19] Sacco RL Kasner SE Broderick JP Caplan LR Connors JJ Culebras A. An updated definition of stroke for the 21st century: a statement for healthcare professionals from the American Heart Association/American Stroke Association. Stroke. (2013) 44:2064–89. 10.1161/STR.0b013e318296aeca 23652265 PMC11078537

[20] Walter K. What is acute ischemic stroke? J Am Med Assoc. (2022) 327:885. 10.1001/jama.2022.1420 35230392

[21] Chen S Zeng L Hu Z. Progressing haemorrhagic stroke: categories, causes, mechanisms and managements. J Neurol. (2014) 261:2061–78. 10.1007/s00415-014-7291-1 24595959 PMC4221651

[22] Czap AL Sheth SA. Overview of imaging modalities in stroke. Neurology. (2021) 97:S42–51. 10.1212/WNL.0000000000012794 34785603 PMC11418094

[23] Abdalkader M Siegler JE Lee JS Yaghi S Qiu Z Huo X. Neuroimaging of acute ischemic stroke: multimodal imaging approach for acute endovascular therapy. J Stroke. (2023) 25:55–71. 10.5853/jos.2022.03286 36746380 PMC9911849

[24] Tao C Nogueira RG Zhu Y Sun J Han H Yuan G. Trial of endovascular treatment of acute basilar-artery occlusion. N Engl J Med. (2022) 387:1361–72. 10.1056/NEJMoa2206317 36239644

